# Pressure Effect on the Rheological Behavior of Highly Filled Solid Propellant During Extrusion Flow

**DOI:** 10.3390/polym17152003

**Published:** 2025-07-22

**Authors:** Jun Zhang, Wei Zheng, Zhifeng Yuan, Junbo Chen, Jiangfeng Pei, Ping Xue

**Affiliations:** 1 College of Mechanical and Electrical Engineering, Beijing University of Chemical Technology, Chaoyang District, Beijing 100029, China; zmz171688@163.com; 2 Xi’an Modern Chemistry Research Institute, 168 Zhangba East Road, Xi’an 710065, China; zhei_wei035991@163.com (W.Z.); yxfllx@163.com (Z.Y.); cjb85_@126.com (J.C.); pjfpmt@163.com (J.P.)

**Keywords:** solid propellants, rheology, pressure, highly filled composites

## Abstract

Currently, the shear-extrusion behavior of solid propellants (SPs), which comprise a significant volume fraction of micro-/nanoscale solid particles (e.g., octogen/HMX), nitroglycerin as a plasticizer/solvent, nitrocellulose as a binder, and other functional additives, is still insufficiently understood. While the rheology of highly filled polymers has been extensively documented, the rheological behavior of SPs within the practical processing temperature range of 80–95 °C remains poorly understood. This study investigated, in particular, the pressure dependence of the viscosity of SPs melts during steady-state shear flow. Steady-state shear measurements were conducted using a twin-bore capillary rheometer with capillary dies of varying diameters and lengths to explore the viscosity dependence of SPs. The results reveal that interface defects between octogen particles and the polymer matrix generate a melt pressure range of 3–30 MPa in the long capillary die, underscoring the non-negligible impact of pressure on the measured viscosity (*η*). At constant temperature and shear rate, the measured viscosity of SPs exhibits strong pressure dependence, showing notable deviations in pressure sensitivity (β), which was found to be greatly relevant to the contents of solvent and solid particles. Such discrepancies are attributed to the compressibility of particle–particle and particle–polymer networks during capillary flow. The findings emphasize the critical role of pressure effect on the rheological properties of SPs, which is essential for optimizing manufacturing processes and ensuring consistent propellant performance.

## 1. Introduction

Solid propellants (SPs), a typical class of energetic materials with polymer binders and a large proportion of solid particles as fillers, have become the mainstream energy source for barrel weapons, rockets, and spacecrafts to launch projectiles [1,2]. Currently, mold extrusion of SPs with controllable progressive combustion is widely preferred for achieving high-accuracy geometry and ballistic performance in barrel weapons. However, SPs are intrinsically sensitive to factors including temperature, friction, impact, and pressure, etc. In this case, the flow status of SP melt during screw extrusion is critical for achieving security assurance. Unfortunately, the flow behaviors of SPs within an extruder are rarely reported. Previous works mainly focused on understanding the constitutive model of SPs with varying formulations [3,4]. Thus, the rheological properties of such materials under sensitive factors are insufficiently understood to optimize their processing.

In addition, precise control of burning surfaces with the desired geometry is generally required for SP products to achieve progressive combustion. Therefore, the one-step extrusion of SPs without any additional turning process is important; this is also dependent on their rheological properties [5,6]. Such extrusion of SPs can be conducted through a mold, including one or more mandrels under a certain melt pressure. Meanwhile, owing to the pressure sensitivity and viscous dissipation, the melt pressure during the extrusion is normally less than ~30 MPa [7,8,9]. With the rapid advancements in the upgrading of weapon systems, 3D printing technology has emerged in the processing of SPs to fabricate advanced architectures [5,10,11]. This novel technology can be employed to produce micro-power sources, micro-initiators, and micro-propulsion systems [11,12]. It should be noted that the demand for SP products has shifted towards complex structures and controllable energy release, which also requires an insight into their rheological behavior.

The extrusion of SPs is usually performed by using a short screw under a relatively low temperature (~80–95 °C) and relatively high pressure to form dense explosive grains. On the one hand, great efforts have been paid to develop high safety guarantees in the traditional extrusion of energetic materials [7,8,9,13,14,15,16,17,18]. Researchers have strived to develop novel compositions for the extrusion of SPs with a combination of safety and combustion properties. On the other hand, optimized technology or equipment for processing SPs is required. However, the rheological properties, especially in melt processing conditions under certain pressure and temperature restraints, are rarely reported. The rheological properties of SPs during mixing and extrusion are considered as key factors to enhance product quality. For instance, the compactness and uniformity of the diameter as well as the roundness of columnar products are of great significance for achieving the expected combustion performance [19]. 

It has been well documented that SPs contain a considerable amount of solid microp–articles, which are indicated in the name of oxidizers and active metal fuels [20,21,22,23,24], such as ammonium perchlorate (AP) and aluminum, magnesium, carbon nanomaterials, and boron, etc. In this case, the flow characteristics of highly filled SPs during extrusion processes are certainly influenced by their compositions [25,26,27,28]. For those highly filled polymer composites, a notable liquid–solid transition will be observed when the concentration of solid particles is above a specific threshold [15]. This transition has been attributed to the intrinsic network structure formed by the solid particles themselves. Similarly, the agglomeration or network formed by the solid particles within the NC solution can also alter the flow behaviors of SPs, which is crucial for optimizing the processing of SPs and ensuring the desired geometries. For highly filled polymeric composites, Sarvestani et al. [29,30] proposed a foundation theory for the creation and disintegration of rigid particle networks embedded within polymer matrices. The particle–particle networks were proposed to be pivotal in determining the viscoelastic properties of highly filled molten systems. Physical interactions among particles, primarily van der Waals forces, induce elastic responses within the particle network. Conversely, particle mobility and network disruption give rise to viscous responses. The viscoelasticity of highly filled polymer nanocomposite melts fluctuates based on the inter-particle distance and the physical interplay between the particle surface and the polymer matrix [31,32]. Moreover, Kourki et al. [33] categorized highly filled polymer composites into three networks: a particle–particle network; a particle–chain network; and a chain–chain entanglement network. When the content of fillers is above the percolation threshold, the particle network enhances the tendency to exhibit a shear plateau effect and liquid–solid transition at a low shear rate [34,35].

The extrusion behavior of highly filled polymer composites could be influenced by pressure [36,37,38,39,40,41,42,43,44,45,46,47], temperature, and shear rate, etc. However, its influence on the viscoelasticity of SPs has rarely been investigated. In order to gain an insight into the flow behavior of SPs during an extrusion process, finite element analysis can be performed. However, accurate simulation results rely on an exact constitutive model [48,49]. Therefore, it is essential to understand the rheological properties of SPs in advance and then to establish a suitable processing routine for solid propellant materials. The pressure exhibits a significant effect on viscosity (*η*), which cannot be ignored in numerical simulations. This viscosity–pressure behavior of SPs is mainly attributed to the compressibility of particle–particle networks and particle–polymer networks during flow. 

Due to the lack of understanding of the pressure-dependent rheological behavior of SPs during extrusion and the restriction of extrusion efficiency [50], an experimental investigation of the pressure dependence of SPs melt viscosity was performed in this study. Inspired by the references of [30,37,51], the rheological properties of SPs could vary greatly from those regularly reported owing to the highly filled solid particles. In this study, therefore, capillary rheometry was employed to investigate the viscosity evolution of SPs during capillary extrusion. Four samples with varying solid filler concentrations and solvent contents were prepared to compare the impact of filler loadings on viscosity. A self-designed twin-bore capillary rheometer was used to measure the steady shear viscosity of SPs across a range of temperatures, with a particular emphasis on pressure-induced deviations in viscosity. 

## 2. Materials and Characterizations

### 2.1. Materials and Formulations

SPs are composed of nitrocellulose, nitroglycerin, and solid particles with minor catalyst, plasticizes, and other additives, as listed in Table 1. Nitrocellulose (NC, ω(N) ~12.0 wt.%) and nitroglycrin (NG) were provided by YiBin North Chuan’an Chemical Industry Co., Ltd., China. Octogen powders (D50, ~22.4 μm) were supplied from Gansu Yinguang Chemical Industry Co., Ltd., Baiyin, China. Plasticizer was purchased from Shanxi Northern Weapons Machinery Co., Ltd., Yongji, China. Industrial-grade ballistic stabilizers were produced by Yunan Micro Powder Factory, China. Here, both the NG/NC ratio and the solid particle content were identified as the key experimental factors when determining the rheological properties of solid propellants (SPs). Specifically, sample S1 had an NG:NC ratio of 0.80, whereas samples S2, S3, and S4 exhibited an increased ratio of 1.17. Samples S3 and S4 incorporated varying solid particles, reaching 25 wt% and 45 wt%, respectively.

### 2.2. Preparation of Samples and Characterizations

#### 2.2.1. One-Pot Compounding of SPs

The mixture of these components was first mixed in water at 50–60 °C for several hours, and were then calendered using a laboratory-scale self-developed calender at approximately 90 °C. The roller’s rotation speed was set to 10–15 revolutions per minute (rpm), and the obtained sheets were granulated into small particles. After calendaring out the most water, the samples should be completely dried in a vacuum oven under a relatively low temperature.

#### 2.2.2. Twin-Bore Capillary Rheometer

**Figure 1:**
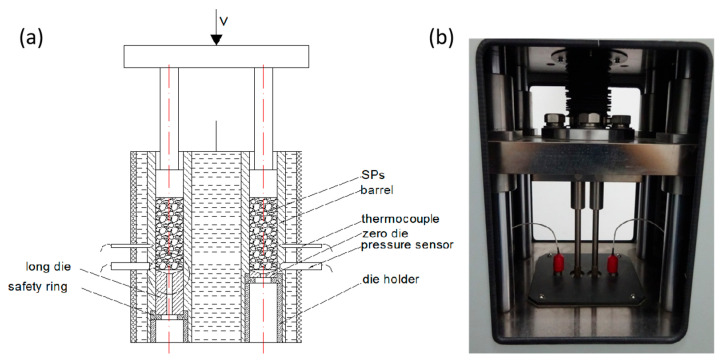
(**a**) Mechanistic illustration of the self-developed twin-bore capillary rheometer and (**b**) a picture of the device.

A self-made twin-bore capillary rheometer was adopted in this experiment and the typical Power-law model was employed to calculated the viscosity, as shown in Figure 1. The barrel of this capillary rheometer is heated by hot water. Except for the special dies and the safety rings, it contains a regular squeezing system with two pistons driven by the same servo motor. Capillary dies with varying diameters *D* = 2.5, 3.0, 3.5, and 4.0 mm and varying lengths of 10, 15, and 20 mm were used. All the capillary dies have the same die entry angle of 180° and a aspect ratio of 2–8.

Compared with a single-bore capillary rheometer, the twin-bore capillary rheometer is fitted with an additional orifice die of effectively zero length (~1 mm). This twin-bore capillary rheometer is a piston-driven, constant-speed capillary rheometer. According to the achievable flow rate and die diameter, the plunger speed allows for apparent shear rates ranging from 10^−1^ to 10^2^ s^−1^ with an allowed pressure. It should be noted that the maximum shear rate cannot be arbitrarily set but is mainly determined by the melt flow rate at the critical pressure value. A threshold value is set for the capillary rheometer when running the tests. For those samples with a high flowability, the achievable flow rate during capillary shear flow could be higher than those with a low ease of flow. This is because samples that are more difficult to flow often require a higher pressure when flowing in the capillary die. The melt temperature can be controlled within ±0.5 °C of the set values of the barrel. Before testing, the SP melt was subjected to pre-compression to extrude the sample from exit to ensure a compacted state of the SPs in the barrel. The equilibrium mode was carried out by the pressure stability, with a pressure deviation of 0.5%. In particular, when the melt pressure exceeds the limited value, which is designated by the safety ring, the pistons will be withdrawn and the extrusion process will be stopped automatically. For a single measurement that demonstrated an increase in discontinuous pressure, the results needed to be discarded and the measurement was repeated. When the relative deviation between the two tested results is lower than 5%, the mean value is taken as the result of the single test. In this case, each sample under certain conditions was tested at least five times and the median data were selected for comparison and discussion.

#### 2.2.3. Scanning Electron Microscopy

The morphologies of both samples were detected through scanning electron microscopy at an accelerating voltage of 15 kV (SEM 5000, Guoyi Quantum Technology (Hefei) Co., Ltd., Hefei, China). Varied magnifications of the surface characteristics of SPs were taken. The fractured surfaces were obtained by manually breaking the extruded propellant rod. In particular, the distribution state of solid particles, as well as the NCs, was detected. All the surfaces were coated by sputtered Au before the SEM observation.

## 3. Results and Discussion

### 3.1. Principle of the Capillary Rheometer

According to the theoretical assumptions of traditional capillary viscosity testing, the apparent shear stress $\tau_{W}$ and the corrected shear rate $\gamma_{W}^{N}$ can be inferred from the stress equilibrium as

(1) $$\tau_{W}=\frac{D(P_{I}-P_{O})}{4L},$$

where *L* is the length of the capillary die, *D* is the diameter of the capillary channel, *P_I_* is the melt pressure at the entrance and *P_O_* is the outlet pressure at the capillary exit. Here, the pressure drop within the capillary die is measured, which does not require a further theoretical correction.

(2) $${\dot{\gamma }}_{W}^{N}=\frac{32Q}{\pi D^{3}}=\frac{8D_{0}^{2}v_{0}}{D^{3}},$$

where *Q* is the volume flow rate, *D*_0_ is the diameter of the barrel, and *v*_0_ is the speed of the piston during testing. In addition, according to the Rabinovich equation, the true shear rate at the wall can be given as

(3) $$\dot{\gamma }={\dot{\gamma }}_{W}^{N}(\frac{3}{4}+\frac{1}{4n}),$$

where *n* is the non-Newtonian flow index for polymeric fluids, which can be calculated by

(4) $$n=\frac{d(\ln\tau_{W})}{d(\ln\gamma_{W}^{N})}.$$

According to the typical Power-law model, the measured shear viscosity η can be obtained by Equation (5), based on the Rabinowitsch correction.

(5) $$\eta =\frac{\tau_{W}}{{\dot{\gamma }}_{W}^{N}}=\frac{D^{4}\cdot \Delta P\cdot n}{8D_{0}^{2}\cdot v_{0}\left(3n+1\right)}.$$

It should be noted that the above principle is built without being assigned to a specific constitutive model, and can therefore be regarded as an universal method for steady and continuous flow. This viscosity calculation principle was initially developed for homogeneous fluids under specific theoretical assumptions. For the twin-bore capillary rheometer, the entrance pressure drop was experimentally removed, which currently becomes a general strategy for capillary viscosity measurement. Based on the previous studies [7,9,52], highly filled polymer composites or even SPs were also found to be well fitted by the Power-law model. Therefore, the aforementioned principle may serve as an effective method to characterize the nonlinear viscoelasticity of highly filled solid propellants (SPs), but the influence of process parameters on the results could be varied. Under a constant shear rate, the measured shear viscosity of highly filled composite melts increases significantly with pressure. Compressible materials are more sensitive to pressure than non-compressible materials, such as those of a similar composition filled with particles. Although this porosity exceeds the levels typically documented in classical polymer physics, the Doolittle theory remains applicable for evaluating melt compressibility, as described in Equation (6).

(6) $$V=V_{0}+V_{f}$$

where *V* is the whole volume of the composite melt, *V*_0_ is the actual volume of the material, and *V_f_* is the void volume, i.e., the porosity. The Doolittle equation proposed an empirical expression equation for the viscosity calculation based on the relationship between *V_f_* and viscosity,

(7) $$\eta =K\exp(NV_{0}/V_{f}),$$

where *η* is shear viscosity and *K* and *N* are constants. This free space within the composites must be compressed as the pressure of the polymer melt increases, which definitely affects the flow behavior of the SPs. Therefore, the measured apparent viscosity of the melt normally exhibits an increasing trend with an increase in pressure.

### 3.2. Dependence of Shear Viscosity on Temperature

**Figure 2:**
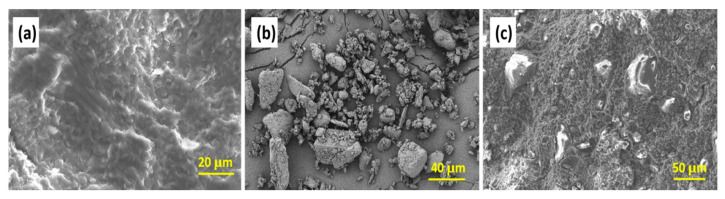
SEM observations of (**a**) NG/NC polymeric matrix, (**b**) octogen solid particles and the obtained (**c**) NG/NC/octogen composites.

Figure 2 illustrates the morphological evolution of SPs before and after the incorporation of solid particles (e.g., energetic octogen), which was obtained by SEM. The cross-sectional morphology of the nitrocellulose (NC)–nitroglycerin (NG) blend (with NC fully dissolved in NG) exhibits pronounced roughness (Figure 2a), accompanied by complex fracture patterns and multiple shear bands. Upon introducing a high volume fraction of solid particles, interface defects arise due to poor compatibility between the particles and the matrix, thereby hindering the complete densification of SPs during extrusion. This results in significant compressibility, leading to flow instability under pressure when the SP is in a molten or highly elastic state. However, the pressure-induced flow might include interface slip behavior, introducing errors in pressure drop measurements.

**Figure 3:**
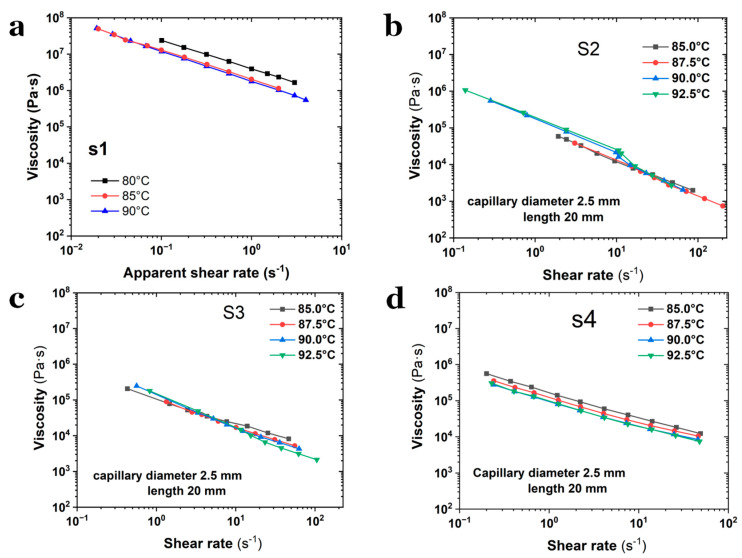
Dependence of shear viscosity on temperature for (**a**) S1, (**b**) S2, (**c**) S3 and (**d**) S4, respectively.

Viscosity-shear rate curves were measured at varying temperatures through the capillary shear mode, as depicted in Figure 3. First, given the diverse compositions of the selected SPs, steady-state shear measurements were conducted to characterize their rheological dependence on melt temperature, which was preferred to be in the range of 80–95 °C to accommodate differences in flowability in practice. Sample S1, which has the lowest NG/NC ratio (0.80), exhibits the highest viscosity (~10^8^ Pa·s), suggesting that the solvent content significantly influences melt extrudability. For samples without octogen particles, the viscosity of S1 displays far greater deviations compared to S2, and even exceeds those of S3 (which contained fewer octogen particles). The high viscosity of S1 could render its melt extrusion through capillary dies with diameters < 5.0 mm extremely challenging. Specifically, S1 could not be extruded through a 2.5 mm die at higher shear rates, as its melt pressure exceeded the safety threshold value, preventing the acquisition of a continuous shear rate range. Consequently, a 5 mm capillary die was employed to measure the viscosity of S1. In contrast, samples S2–S4 were tested using a 2.5 mm die to achieve a broader shear rate range.

The results shown in Figure 3 reveal that samples S3 and S4, which contain octogen solid particles, exhibit lower viscosities compared to S1 and S2. For instance, at 85 °C and a shear rate of 1.0 s^−1^, the viscosity of S1 (measured using a 5 mm capillary die) is approximately 1.3 × 10^7^ Pa·s, but it drops to ~2 × 10^6^ Pa·s at higher temperature. In contrast, S3 and S4 show viscosities of ~1.2 × 10^5^ Pa·s and ~1.7 × 10^5^ Pa·s, respectively. Notably, S4, which has the highest octogen content, exhibits a much lower viscosity than S1, suggesting that this reduction is not solely due to shear effects and may reflect non-intrinsic rheological behavior.

Overall, all measured viscosity curves display typical shear-thinning behavior, and their flow and viscosity profiles indicate that their flow behaviors during processing can be adequately described by the Power-law constitutive model. While viscosity decreases with increasing temperature, the temperature effect is less pronounced compared to the shear rate effect. At low shear rates, the flow of SPs melt becomes unstable, leading to inconsistent viscosity patterns. Additionally, the temperature dependence of viscosity seems to diminish as the NG content increases.

### 3.3. Flow Behaviors

**Figure 4:**
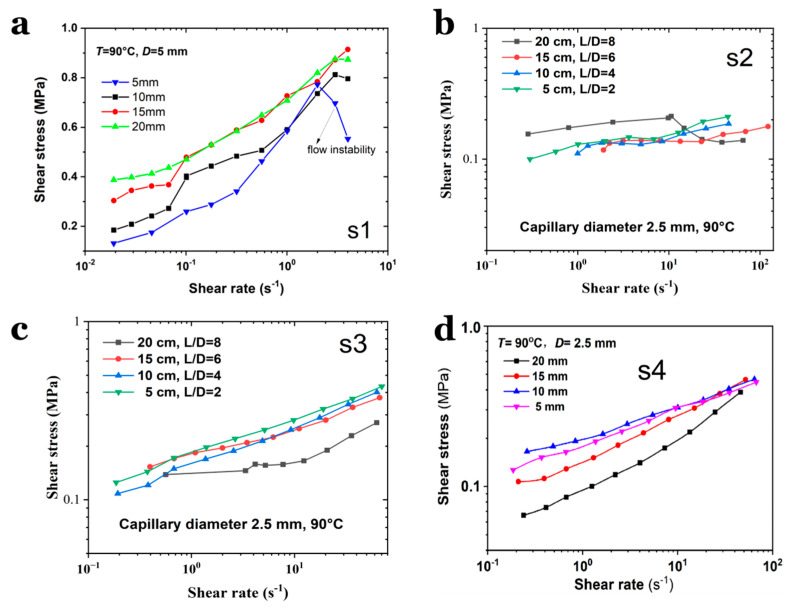
Flow curves tested under varied capillary die length for (**a**) S1, (**b**) S2, (**c**) S3 and (**d**) S4, respectively.

The flow curves shown in Figure 4 reveal distinct stress-strain rate behaviors across the samples. When a sample lacks octogen solid particles, it behaves as a concentrated polymeric solution (e.g., NC dissolved in NG, as seen in S1). The shear stress of S1 during capillary flow increases rapidly with a rise in the shear rate. At the same shear rate, the shear stress experienced by S1 melt in a longer capillary die is lower than in a shorter die, likely due to reduced frictional losses in short channels. In this study, the low shear rates during capillary flow imply that the shear stress magnitude represents the minimum driving force required to extrude the melt through the capillary. It should be noted that all the flow curves of S1–S4 clearly demonstrate flow instabilities, even at a low shear rate, which implies the inhomogeneity of the samples, e.g., compressibility. Moreover, such unstable flow behavior seems to be more remarkable in the small capillary dies, suggesting a geometrical dependence of the shear flow of SPs.

The flow behaviors of the four samples differ significantly when passing through the varying capillary dies. For S4, the presence of numerous solid particles reduces the frictional resistance between the bulk material and the channel wall, enabling flow at lower pressures and even inducing a viscous–slip transition at low flow velocities. For samples with a high NG content and no solid particles (e.g., S2), the flow curves are smoother, indicating enhanced shear flow in the capillary die, even for SPs with minimal octogen particles (e.g., S3). This means that if solid particles were present at the shear interface (e.g., between the extrudate and the barrel wall), their frictional resistance against the capillary wall would be reduced. The flow curve of S1 (seen in Figure 4) aligns with viscoelastic fluid behavior in capillary channels, requiring a higher pressure gradient for longer channels to maintain steady flow. In S2, the higher NG content facilitates shear flow in the capillary, even for SPs with a low octogen content (e.g., S3). For S3 and S4, the pressure drop could be concentrated near the die entrance and the slip effects cannot be ignored. This results in a higher pressure drop (i.e., shear stress) in short dies compared to long dies.

It is important to note that the above conclusions were derived under the assumption of negligible wall slip. If the wall slip were significant, the flow pattern of S1 could resemble that of S4. The experimental results were obtained at low shear rates due to extremely low flow velocities. As the shear rate increased, the flow curves of S4 in capillary dies with different lengths gradually converged, with shear stress becoming consistent at shear rates above 100 s^−1^. In contrast, for S1–S3, the flow curves diverged at higher shear rates, suggesting distinct shear-dependent behaviors. These findings indicate that, for the same sample, the measured shear stress varies with the geometric dimensions of the capillary die, even under identical testing conditions (i.e., different shear stresses at the same shear rate). However, no clear pattern of shear stress variation with capillary die diameter was observed, suggesting a weak dependence of flow behavior on die diameter. Notably, S4 exhibited higher shear stress in larger capillary dies, indicating that melt pressure and sample composition significantly influence the flow behavior of SPs within capillary channels.

### 3.4. Viscosity of SPs on the Geometry of Capillary Die

**Figure 5:**
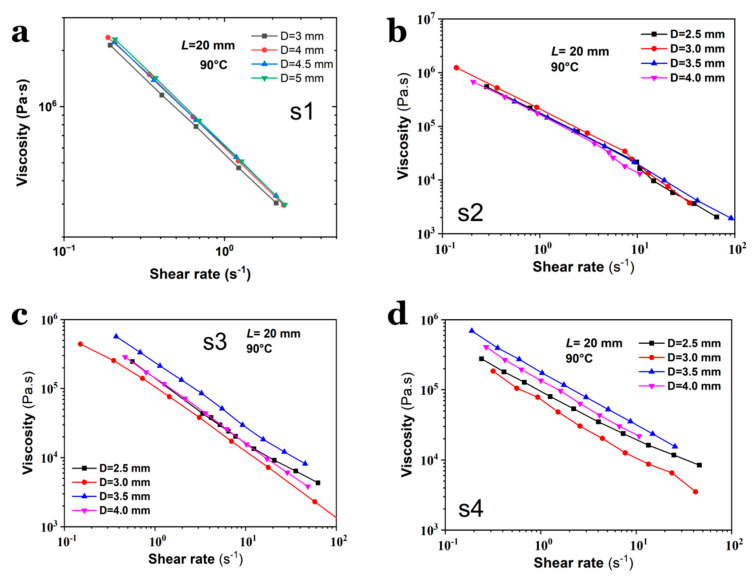
Flow curves of (**a**) S1, (**b**) S2, (**c**) S3 and (**d**) S4 measured from the capillary die with varying diameters and a constant length of 20 mm, respectively.

**Figure 6:**
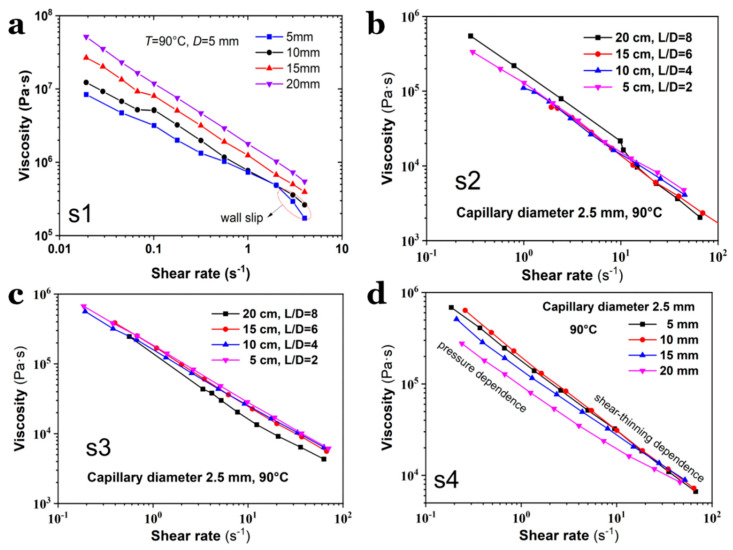
Viscosity curves of (**a**) S1, (**b**) S2, (**c**) S3 and (**d**) S4 measured from varied capillary dies, respectively.

To investigate the viscosity evolution of SPs measured using capillary dies with varying diameters, the flow curves of S1–S4 were characterized using dies with an identical length of 20 mm but varying diameters of 2.5~4.0 mm, and dies with an identical diameter but varying lengths, as shown in Figure 5 and Figure 6. All the viscosity measurements were conducted at 90 °C. Compared to S3 and S4, S1 and S2 exhibit less dependence of shear viscosity on the capillary die diameter (Figure 5a,b), suggesting that the presence of octogen particles induces distinct shear behaviors during capillary flow (Figure 5c,d). Interestingly, the results in Figure 6 reveal visible differences in the measured viscosity, which are strongly dependent on the aspect ratio of the capillary die. First, the shear rate range for S1 is narrower than that for S2–S4, differing by approximately one order of magnitude. Second, S1 attains its highest viscosity in the 20 mm-long die, whereas the viscosity of S4 peaks in the 5 mm-long die. For S2 and S3, the viscosity curves are nearly identical, indicating that the shear stress in long dies closely matches that in short dies. Compared with the flow curves shown in Figure 4, the viscosity curves in Figure 6 reveal higher stability. This indicates that the flow instabilities could mainly result from the entrance flow.

The discrepancies in viscosity curves obtained by using capillary dies with different aspect ratios (*L*/*D*) suggest that the viscosity of the same material may vary even when measured under identical conditions (e.g., temperature, shear rate). The underlying reasons for this viscosity deviation remain unclear. To address this issue, we propose that melt pressure within the capillary die is a key factor influencing viscosity, particularly for SPs containing a high volume fraction of solid particles. The pressure generated in dies with varying aspect ratios differs significantly, leading to experimental viscosity variations that can be explained by the free volume principle.

The pressure sensitivity of viscosity varies across materials. For instance, the viscosity curves of S4 diverge at low shear rates but converge at a high shear rate region, indicating that shear rate dominates over pressure in determining the flowability of S4 melt at high shear. Therefore, the pressure effect during capillary flow must be carefully considered when analyzing the rheological properties of SPs, especially those with high solid particle content.

### 3.5. Viscosity of SPs on Melt Pressure

**Figure 7:**
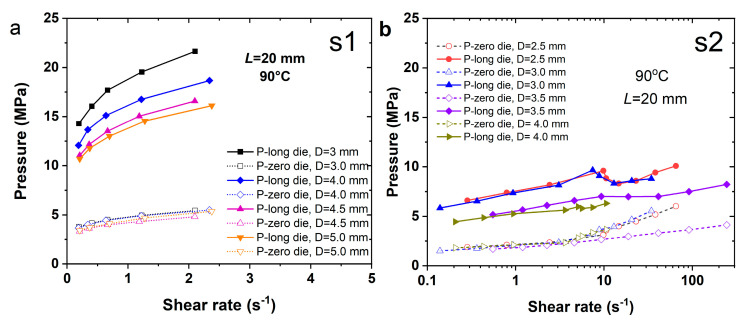

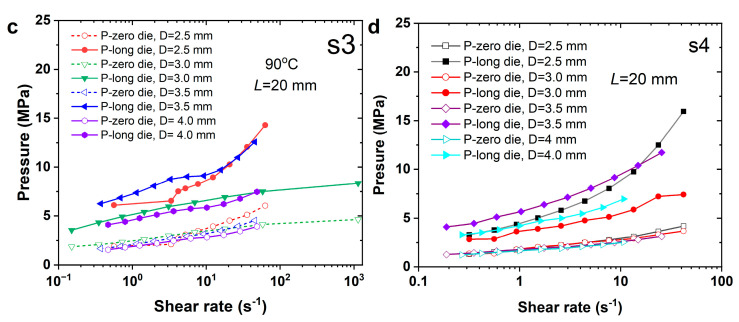
Curves of melt pressure measured in both the long die and the zero die for (**a**) S1, (**b**) S2, (**c**) S3 and (**d**) S4, respectively.

Figure 7 displays pressure vs. shear rate curves measured using long/short capillary dies in a twin-bore capillary rheometer. All the dies have a fixed length of 20 mm but vary in diameter. The results reveal that S1 melt requires significantly higher pressure to maintain the extrusion flow, i.e., showing a much higher pressure drop. In contrast, S2 exhibits minimal pressure differences. The shear flow of S1 melt is confined to a narrow shear rate range (≤10 s^−1^); beyond this, the melt pressure risks exceeding the safety threshold, indicating poor flowability. Compared to S2 and S3, the melt pressure of S1 in the long die increases more sharply with shear rate, while the short die shows a smoother pressure rise, suggesting an enhanced pressure drop. Over the same shear rate range, S2 and S3 exhibit gradual pressure increases in the long die. These discrepancies stem from the pressure-dependent viscosity of polymeric melts. Notably, the pressure in smaller dies exceeds that in larger dies by ~3–5 MPa at identical shear rates, as reflected in the long/short die measurements. The large pressure drop of S1 indicates that the shear flow of S1 (mass ratio NG:NC ~0.80) requires a much higher pressure drop to overcome the flow resistance. Even though S1 is free of solid particles, the melt pressures revealed in both barrels are still much higher than those indicated in S4.

For S4, the melt pressure measured at identical temperatures and capillary die lengths is not only lower than that of S1 but also relatively lower than those of S2 and S3. Specifically, the melt pressure in the long die at a shear rate of 1 s^−1^ is only ~3–6 MPa, and even slightly lower than the pressure of S1 in the short die. The melt pressure in the short die for S4 remains consistent, while the pressure in the long die clearly increases with the shear rate. However, there is no longer any correlation with the die diameter—a behavior distinct from the flow characteristics of S1.

The flow pattern of S1 in the capillary channel is relatively stable, with observed differences in measurements across dies of varying aspect ratios and diameters. As given in Equations (1) and (2), the shear stress magnitude depends on the die diameter, yet the viscosity of S1 (Figure 5a) shows minimal variation across dies of different diameters. In contrast, the pressure vs. shear rate curves of S4 (Figure 7d) exhibit a J-type pattern, indicating pronounced deviations at higher shear rates. Overall, Figure 7 underscores a strong correlation between melt pressure and viscosity, particularly for the highly filled S4 melt, where melt pressure dominates viscosity determination.

To further quantitatively evaluate the measured shear viscosity dependence on their melt pressure, the Barus equation is employed [47], as shown in Equation (8).

(8) $$\eta =\eta_{P0}e^{\beta \left(P-P_{0}\right)},$$

where *η* (Pa·s) is the practically measured shear viscosity under pressure *P*, *η*_P0_ is the shear viscosity at atmospheric conditions, *β* is the pressure coefficient (GPa^−1^), *P* is the characteristic melt pressure (GPa) during flow, and *P*_0_ is the pressure around the flow outlet. In this study, the pressure *P*_0_ is regarded as zero and the viscosity *η*_P0_ should be constant for a given shear rate under given temperature. Then, Equation (8) can be changed to

(9) $$\ln\eta =\beta P+\ln\eta_{P0}.$$

**Figure 8:**
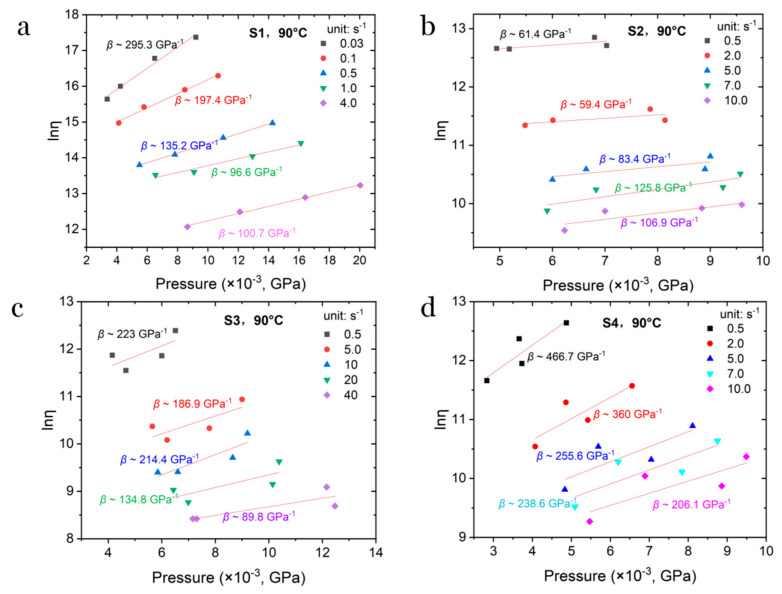
Fitted curves of ln*η* vs. *P* and the obtained *β* for (**a**) S1, (**b**) S2, (**c**) S3 and (**d**) S4 at 90 °C, respectively.

Therefore, Equation (9) predicts that, for a given shear rate, *β* can be calculated by the slope rate of the plot of logarithm shear viscosity ln*η* versus *P*, thus enabling *β* to be calculated. In this study, the pressure measured from the long die was used as the characteristic melt pressure *P*. Generally, the value of *β* indicates the degree of dependency of viscosity on the pressure. Figure 8 shows the evolution of the *β* value with the shear rate and the composition of the sample. Compared with Figure 8a,b, it can be found that the shear viscosity of S1 reveals a much higher sensitivity to pressure. After incorporating more solvent (i.e., NG), this dependence was greatly reduced. However, with in the presence of solid particles, e.g., S3 and S4, the pressure sensitivity of viscosity increased remarkably, especially for S4. In addition, the *β* value decreases with the increase in the shear rate, suggesting an enhanced shear-thinning effect. In addition, compared with S1 and S3–S4, S2 shows a reversed evolution. The *β* of S2 increases with the increase in shear rate, indicating that S2 is more compressible at a higher shear rate.

**Figure 9:**
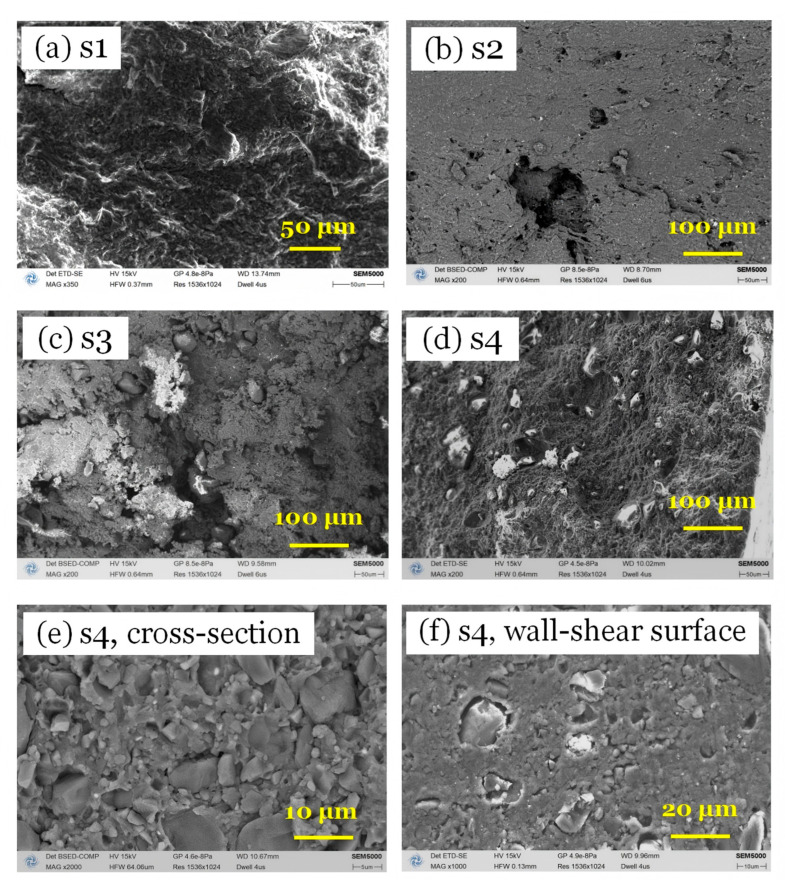
(**a**–**d**) Surface morphology of S1–S4 and (**e**) the dispersion of solid particles and (**f**) the visible interfacial defects within the S4.

To further elucidate the rheological complexity of solid propellants (SPs), their morphologies were compared, as shown in Figure 9. The S1 and S2 samples, lacking octogen particles, exhibit full wall shear flow. Conversely, samples containing solid particles show surface features that prevent full contact with the capillary wall, violating the no-slip boundary condition and complicating the shear behavior. The characteristic whereby solid particles anchored at the extrudate surface reduce the shear surface area and thereby decrease the flow resistance, revealing a reduced viscosity, are shown in Figure 9e,f. In addition, the formed interface defects are compressible under high pressure, indicating a varying pressure sensitivity of viscosity.

## 4. Conclusions

In summary, this study experimentally investigated the effect of melt pressure on the steady-state shear rheological properties of highly filled solid propellants (SPs). Using a twin-bore capillary rheometer, the pressure dependence of viscosity was examined within a temperature range of 80–95 °C, employing capillary dies with varying diameters and lengths. Particular emphasis was placed on the pressure from the long die to elucidate viscosity deviations as well as its sensitivity; this was not considered in the viscosity calculation, where only the pressure drop was taken into account. The results revealed that the presence of substantial solid particles and the associated interfacial defects between particles and polymers induced a melt pressure range of 3–30 MPa in the long die, underscoring the non-negligible pressure dependence of viscosity for SPs. The observed viscosity deviations across different capillary dies (under identical conditions) were analyzed through the free volume principle. Overall, this study highlights the critical role of pressure effects on the rheological properties of SPs, which is essential for optimizing SP production and processing.

## Figures and Tables

**Table 1:** Formulations of samples for rheological measurement (unit: wt.%).

Samples	Components					
	Polymeric Matrix				Solid Particles	
	NC	NG	Centralite	Plasticizer	Catalyst	Octogen
S1	54.7	43.8	1.5	/	/	/
S2	42.5	50	1.5	3.5	2.5	/
S3	32.2	37.8	1.5	3.5	2.5	22.5
S4	23	27	1.5	3.5	2.5	42.5

Remarks: NC, nitrocellulose, C_6_H_7_N_3_O_11_. NG, nitroglycerin, C_3_H_5_N_3_O_9_.

## Data Availability

The original contributions presented in this study are included in the article. Further inquiries can be directed to the corresponding author.
